# Concurrent Validity of Accelerations Measured Using a Tri-Axial Inertial Measurement Unit while Walking on Firm, Compliant and Uneven Surfaces

**DOI:** 10.1371/journal.pone.0098395

**Published:** 2014-05-27

**Authors:** Michael H. Cole, Wolbert van den Hoorn, Justin K. Kavanagh, Steven Morrison, Paul W. Hodges, James E. Smeathers, Graham K. Kerr

**Affiliations:** 1 School of Exercise Science, Australian Catholic University, Brisbane, Queensland, Australia; 2 Movement Neuroscience Program, Institute of Health and Biomedical Innovation, Queensland University of Technology, Brisbane, Queensland, Australia; 3 The University of Queensland, Centre for Clinical Research Excellence in Spinal Pain, Injury and Health, Brisbane, Queensland, Australia; 4 Centre for Musculoskeletal Research, Griffith University, Gold Coast, Queensland, Australia; 5 School of Physical Therapy and Athletic Training, Old Dominion University, Norfolk, Virginia, United States of America; 6 School of Exercise and Nutrition Sciences, Queensland University of Technology, Brisbane, Queensland, Australia; Charité University Medicine Berlin, Germany

## Abstract

Although accelerometers are extensively used for assessing gait, limited research has evaluated the concurrent validity of these devices on less predictable walking surfaces or the comparability of different methods used for gravitational acceleration compensation. This study evaluated the concurrent validity of trunk accelerations derived from a tri-axial inertial measurement unit while walking on firm, compliant and uneven surfaces and contrasted two methods used to remove gravitational accelerations; i) subtraction of the best linear fit from the data (detrending); and ii) use of orientation information (quaternions) from the inertial measurement unit. Twelve older and twelve younger adults walked at their preferred speed along firm, compliant and uneven walkways. Accelerations were evaluated for the thoracic spine (T12) using a tri-axial inertial measurement unit and an eleven-camera Vicon system. The findings demonstrated excellent agreement between accelerations derived from the inertial measurement unit and motion analysis system, including while walking on uneven surfaces that better approximate a real-world setting (all differences <0.16 m.s^−2^). Detrending produced slightly better agreement between the inertial measurement unit and Vicon system on firm surfaces (delta range: −0.05 to 0.06 vs. 0.00 to 0.14 m.s^−2^), whereas the quaternion method performed better when walking on compliant and uneven walkways (delta range: −0.16 to −0.02 vs. −0.07 to 0.07 m.s^−2^). The technique used to compensate for gravitational accelerations requires consideration in future research, particularly when walking on compliant and uneven surfaces. These findings demonstrate trunk accelerations can be accurately measured using a wireless inertial measurement unit and are appropriate for research that evaluates healthy populations in complex environments.

## Introduction

Accelerometry is a reliable and cost-effective alternative for the measurement of gait in various populations. One of the main advantages, compared to conventional optical motion capture systems, is that data can be collected continuously over an extended time period and over long distances [1], [2], as there are fewer restrictions with respect to predefined and often small calibrated spaces [3]. The relatively portable and lightweight design of accelerometers suits them to collection of quantitative gait data in environments where the use of camera-based systems would not be feasible (e.g. within the home environment) [2], [4]. Accelerometers and inertial measurement units (IMU) have been used extensively to assess aspects of postural control during gait in healthy younger [3], [5]–[12] and older adults [13]–[19].

Importantly, previous research has examined the accuracy of the measures derived from these devices against a ‘gold standard’ method (i.e. three-dimensional motion analysis) to evaluate concurrent validity under both strictly-controlled experimental conditions [20], [21] and during dynamic tasks, such as walking [22]–[26]. Of the studies that have evaluated these devices during walking, three were completed on a motorised treadmill [23], [25], [26] and two were completed overground on a firm walkway [22], [24]. Collectively, these studies demonstrated that three-dimensional accelerometers coupled with gyroscopes and IMUs can accurately detect changes in the orientation of the trunk [22]–[25] and lower limb segments [24], [26] while walking on predictable surfaces. Furthermore, Mayagoitia et al. [26] reported that shank and thigh accelerations derived from a motion analysis system and a series of uniaxial accelerometers were no more than 15% different while walking on a treadmill. However, it is important to note that the authors of this study affixed the accelerometers and gyroscopes to rigid aluminium plates on the shank and thigh, which would be expected to influence the damping and frequency properties of the signal differently to techniques that utilise skin-mounted sensors [27]. Despite the existing evidence for the validity of data derived from these devices, it remains unclear whether these findings would be transferable to real-world walking environments with surfaces of different textures, densities and gradients that require constant adjustment of the body's movement patterns to maintain stability.

Although alternating current (AC) coupled accelerometers are known to be insensitive to gravitational accelerations [28], many previous studies have relied on direct current (DC) coupled devices that inherently record both gravitational and movement-related accelerations [7], [12], [13], [18], [20], [21]. As such, a critical factor to consider when evaluating differences in amplitude between accelerations derived from accelerometers and motion capture systems is the most appropriate method for separating these two components of the accelerometer data. Although several methods have been implemented to minimise the influence of gravitational acceleration on accelerometer outputs, each is based on different assumptions and little is known about the comparability of the methods. One method to compensate for the effects of gravity involves subtraction of linear trends from the accelerometer data [29]. This approach, referred to as "detrending", assumes the effects of gravity can be represented by a low frequency component within the acceleration signal. However, research shows that the movement patterns of the head and pelvis are altered when walking on compliant [30] and uneven surfaces [11], [18] and therefore detrending may not be the best method to remove the effects of gravitational acceleration under these conditions. Furthermore, while it is widely accepted that gravitational accelerations can be accounted for by expressing the movement-related accelerations in a frame that takes into account the estimated orientation of the sensor during upright standing, the specific details of this process have not routinely been reported in previous research. Given that most modern IMUs also provide orientation information of the device in relation to a world axis system, it would be possible to use this information to subtract the acceleration due to gravity continuously and directly from the movement-related accelerations [31]. However, it is unclear whether differences in the methods used to compensate for gravitational acceleration would yield different results and potentially influence one's ability to compare acceleration amplitudes presented in different studies.

This research aimed to provide a comprehensive assessment of the; 1) concurrent validity of trunk accelerations derived from a three-dimensional IMU for groups of younger and older adults when walking on firm, compliant and uneven surfaces; and 2) effect of using two different methods to correct for gravitational acceleration; namely, detrending and subtraction using orientation information derived from the IMU. Assessment of concurrent validity of trunk accelerations derived from an accelerometer is of particular importance, given this body segment is often used to evaluate walking stability and falls risk in different populations [7], [8], [11], [16]–[19], [32].

## Methods

### Ethics Statement

The experimental protocol was approved by the Human Research Ethics Committee at the Queensland University of Technology and conducted in accordance with the Declaration of Helsinki.

### Study Population

Twelve healthy older participants aged between 65 and 90 years (mean ± SD; 71.2±4.0 yrs) and twelve healthy younger adults aged between 20 and 25 years (mean ± SD; 22.8±2.0 yrs) gave written informed consent to participate in this study (Table 1). Participants were deemed to be healthy if they were independently living and reported having no existing or recurring medical conditions that adversely affected their balance or mobility (e.g. vestibular disorders, neurological conditions). Prior to recruitment, prospective participants were interviewed over the telephone to discuss their medical history and, prior to data collection, all participants provided a list of their current medications to establish an understanding of their overall health. Participants were excluded if they had any known medical condition that would affect their balance or mobility (e.g. vestibular disorder, neurological impairment), had recent or recurrent history of surgery or musculoskeletal injury or were unable to ambulate independently without the use of a walking aid.

**Table 1:** Participant demographics.

	Older Adults (n = 12)	Younger Adults (n = 12)	Test	*p*-value
***Demographics***				
Age (years)	71.2 (4.0)	22.8 (2.0)	1	**<0.001**
Gender (male)	6 (50%)	6 (50%)	2	1.00
Height (cm)	169.2 (11.8)	174.2 (9.3)	3	0.26
Mass (kg)	73.9 (14.1)	72.0 (11.4)	3	0.71
Body Mass Index (kg/m^2^)	25.6 (2.5)	23.6 (2.4)	3	**0.05**

Data are mean (SD) or absolute numbers and percentages. Test 1 = Kruskal-Wallis Test; Test 2 = χ^2^ test; Test 3 = one-way ANOVA.

### Apparatus

**Figure 1:**
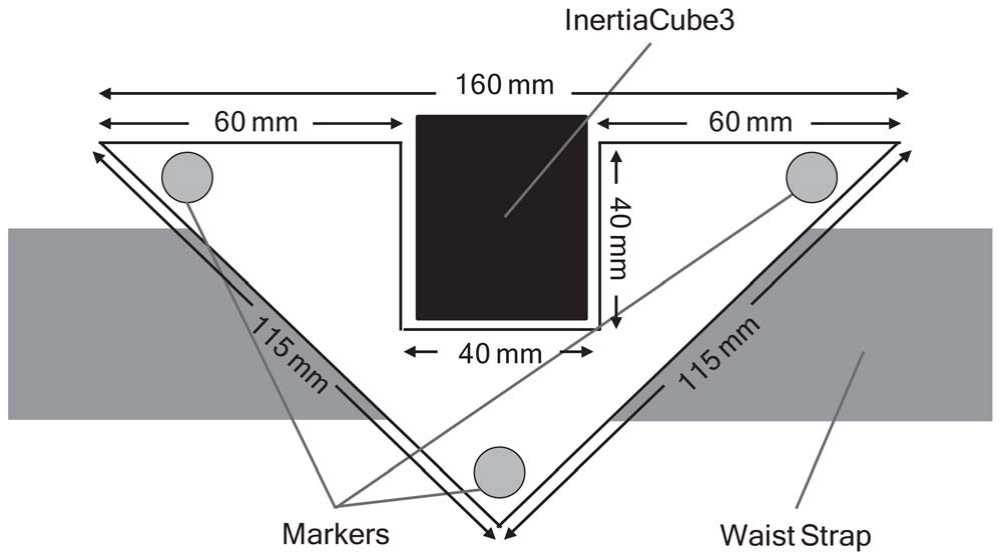
The three reflective markers attached to the custom-built rigid body positioned around the InertiaCube3 Inertial Measurement Unit.

An InertiaCube3 tri-axial IMU (InterSense Inc., Bedford MA, USA) was attached to the skin overlying the spinous process of the 12^th^ thoracic vertebra to measure trunk accelerations during walking. The IMU was attached to the skin using Tesa 4965 polyester double-sided tape (Tesa Tape Inc., Charlotte, NC, USA) and firmly reinforced using Micropore tape (3 M, North Ryde, NSW, AU). A custom-made rigid body that comprised three 14 mm reflective markers was firmly secured around, but not in contact with (at least 3 mm clearance), the IMU using an adjustable Velcro strap. Lateral slippage of the rigid body was minimised by a firm backing that allowed it to sit neatly against the spinous processes of the spine between the trunk extensor muscles (Figure 1). The rigid body was designed to maximise the capacity of this equipment to closely match the movements of the IMU without restricting the participants' movements.

### Test Procedures

Participants walked barefoot at a self-selected and preferred pace along 3 surfaces; i) a firm surface (L: 870 cm; W: 120 cm); ii) a compliant surface (L: 870 cm; W: 90 cm; H: 3.6 cm); and iii) an uneven surface (L: 960 cm; W: 90 cm; H: 5 cm). The uneven surface comprised a series wooden blocks (L: 10.5 cm; W: 7 cm; H: 2 cm), which were placed at random locations under a layer of foam and artificial turf. Three-dimensional accelerations were collected wirelessly via Bluetooth from the IMU at 100 Hz and interpolated to an effective sampling rate of 200 Hz using a cubic spline function. The three reflective markers on the rigid body were tracked (200 Hz) within the central 4 m length of the walkways by a calibrated eleven-camera motion analysis system (Vicon Nexus; Oxford, UK). The accelerometer recordings were synchronised with the three-dimensional motion analysis data by an event signal triggered by the experimenter during each trial. Three trials were performed on each surface and the order in which the walking surfaces were completed was randomised for each individual.

### Data analysis

Data were analysed using software developed in MATLAB (v.7.13; The MathWorks Inc., Natick, MA, USA). Accelerations from the IMU were derived in relation to the accelerometer's axis system and low-pass filtered at 10 Hz using a bi-directional fourth order Butterworth filter. Two methods were then used to separate the movement-related accelerations from the gravitational acceleration. The first method, the "detrending" method, assumed the gravitational component was a very low frequency component that offset the movement-related accelerations, and accounted for this offset by removal of the best linear fit from the acceleration signal. The second method, the "quaternion" method, used the orientation information provided by the IMU, expressed in quaternions, which were derived from a statistical combination of the internal 3D magnetometer, gyroscope and accelerometer using a proprietary Kalman filter. The gravity vector (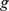) was rotated by the sampled unit quaternions (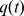), after which the rotated gravity vectors (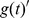) were subtracted from the acceleration vectors at each time point (Equation 1).
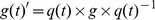
(1)Position data from Vicon were derived from the average position of the three reflective markers and low-pass filtered with a bi-directional fourth order Butterworth filter with a cut off frequency of 10 Hz. Accelerations were derived in relation to Vicon's global coordinate system by differentiation of the average position of the cluster markers twice over time. To enable comparisons in a similar frame of reference, an axis system was created that aligned the orientation of the 3 non-collinear reflective markers on the rigid body with the accelerometer axis system, such that the X-axis was directed forward, the Y-axis was directed to the left and the Z-axis was directed vertically. These accelerations were rotated to the cluster (and accelerometer) axis system using the cluster-generated rotation matrices to provide accelerations that were consistent with the accelerometer's frame of reference. The amplitude of accelerations was calculated as the root mean square (RMS) of the signal with a time window of 0.02 s and averaged across each trial (minimum 3 gait cycles) for the anteroposterior (AP), mediolateral (ML) and vertical (VT) directions separately.

### Statistical Analysis

To evaluate differences between the older and younger participant groups, continuous demographic variables were examined using a one-way analysis of variance (ANOVA) and the degree of association between the categorical variables was assessed with the chi-square (χ^2^) test. To compare the mean RMS AP, ML and VT accelerations derived from the Vicon system and the IMU using both the detrended and quaternion methods (method, 2 levels) on the firm, compliant and uneven surfaces (surface, 3 levels), a repeated measures ANOVA was used. When a statistically significant main effect was identified, the Fisher's least significant difference post-hoc test was used to determine which methods and/or surfaces were significantly different. If one or more of the assumptions of the parametric procedures were violated, the data were analysed using the non-parametric Kruskal-Wallis or Friedman tests.

To evaluate the trial-to-trial reliability of the accelerations derived from the IMU (detrending and quaternion methods) and the Vicon motion analysis system during the three walking trials, ICCs were calculated for the repeated measurements taken on each surface (ICC(2,1), absolute agreement). For this study, ICCs<0.4 were considered to represent poor agreement, values between 0.4 and 0.75 were considered to represent fair to good agreement and values >0.75 represented excellent agreement [33]. The outcomes of this analysis demonstrated excellent reliability for the repeated measures of AP, ML and VT acceleration on the firm and compliant walking surfaces (ICC range: 0.76 to 0.97). Both groups recorded similarly high ICCs for AP and VT accelerations on the uneven surface (ICC range: 0.75 to 0.94), but poorer ICCs for ML accelerations on this surface suggested more trial-to-trial variability in this measure for the older participants (ICC range: 0.39 to 0.52).

Given the excellent trial-to-trial reliability observed, the RMS accelerations for the three trials on each walking surface were averaged and the concurrent validity of the IMU and Vicon systems was appraised using a two-way ANOVA with random effects model. The intraclass correlation coefficient (ICC(2,1), absolute agreement) was calculated as the ratio of the variance between the participants and between the instruments in relation to the relative error [34]. The average bias was calculated as the mean difference between the two instruments (delta) and the 95% limits of agreement were calculated as the standard deviation (SD) of the delta scores multiplied by 1.96 [35]. To test whether the mean difference between the accelerations collected using the IMU and Vicon system (i.e. the bias) differed significantly from zero, a single-sample t-test was used to compare the values against no difference. The standard error of measurement (SEM) across the two instruments was calculated as the pooled SD between the two instruments multiplied by the square root of 1 minus the ICC [36]. The minimum detectable difference (MDD) was calculated as 1.96 multiplied by the square root of 2 times the SEM. To establish whether any biases derived from the IMU was influenced by the age of the participants, the delta scores for the two groups were statistically compared using a one-way ANOVA. Although this was not a primary aim of this study, it was deemed to be important, given that previous research has reported age-related differences in acceleration patterns during walking [15]–[17]. All measures of validity were calculated using a custom function developed in MATLAB [37] and statistical comparisons were conducted using SPSS 21 with the level of significance set at *p*<0.05.

## Results

**Figure 2:**
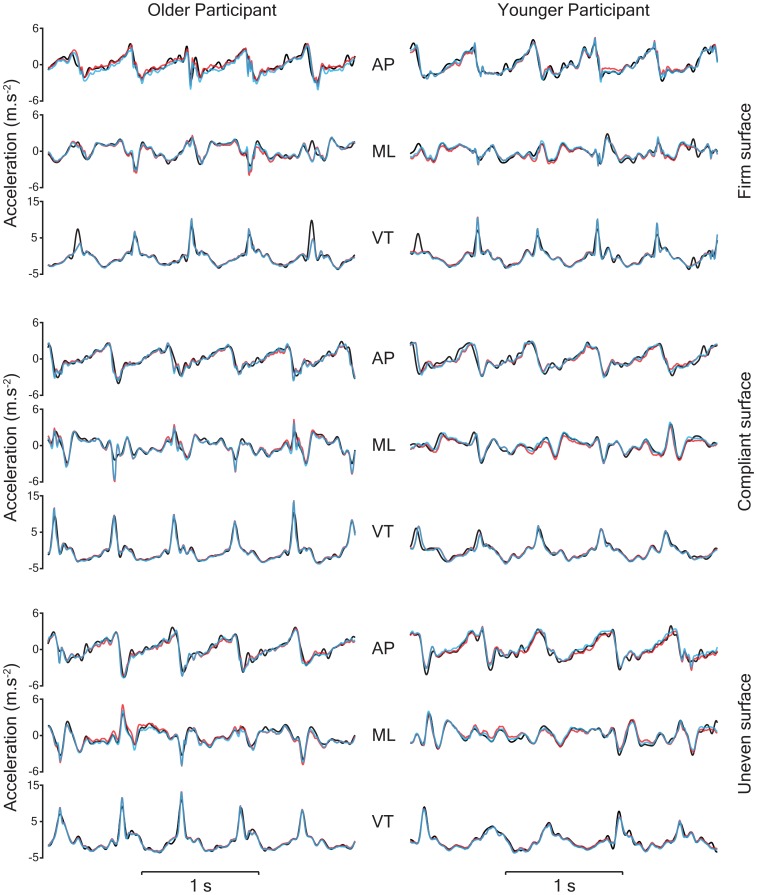
Representative raw three-dimensional trunk accelerations for an older and younger participant collected using the Vicon system (black) and the InertiaCube3 device following gravity compensation using the detrending (red) and quaternion (blue) methods.

Figure 2 provides examples of the AP, ML and VT accelerations derived from the Vicon motion analysis system and the IMU following compensation for gravity (detrending and quaternion methods) while walking on the firm, compliant and uneven surfaces. Average three-dimensional trunk acceleration (RMS) collected via the IMU and Vicon systems for the younger and older participants while walking on the three surfaces are presented in Table 2.

**Table 2:** Root mean square (RMS) accelerations (m.s^-2^) measured by the InertiaCube3 (IC3) and Vicon systems.

			Older Participants (n = 12)			Younger Participants (n = 12)			
			IC3	Vicon	Delta	IC3	Vicon	Delta	Sig.
***Firm Surface***	***Method***	***Axis***							
*RMS Acceleration*	*Detrending*	*AP*	1.18 (0.19)	1.18 (0.21)	0.00 (0.12)	1.32 (0.21)	1.37 (0.22)	−0.05 (0.10)	ns
	*Quaternion*		1.32 (0.22)		0.14 (0.09)	1.49 (0.19)		0.11 (0.12)	ns
	*Detrending*	*ML*	1.18 (0.20)	1.12 (0.18)	0.06 (0.16)	1.09 (0.24)	1.05 (0.23)	0.04 (0.13)	ns
	*Quaternion*		1.19 (0.19)		0.07 (0.13)	1.10 (0.24)		0.05 (0.11)	ns
	*Detrending*	*VT*	1.97 (0.34)	2.00 (0.33)	−0.02 (0.06)	2.08 (0.50)	2.07 (0.46)	0.01 (0.10)	ns
	*Quaternion*		2.00 (0.34)		0.00 (0.06)	2.10 (0.49)		0.03 (0.09)	ns
***Compliant Surface***									
*RMS Acceleration*	*Detrending*	*AP*	1.24 (0.25)	1.31 (0.23)	−0.07 (0.07)	1.39 (0.27)	1.51 (0.26)	−0.12 (0.10)	ns
	*Quaternion*		1.38 (0.25)		0.07 (0.07)	1.54 (0.24)		0.03 (0.08)	ns
	*Detrending*	*ML*	1.14 (0.19)	1.17 (0.19)	−0.03 (0.13)	1.10 (0.23)	1.11 (0.20)	−0.02 (0.08)	ns
	*Quaternion*		1.16 (0.17)		−0.02 (0.07)	1.10 (0.23)		−0.01 (0.07)	ns
	*Detrending*	*VT*	2.12 (0.31)	2.19 (0.34)	−0.08 (0.06)	2.25 (0.52)	2.33 (0.51)	−0.08 (0.08)	ns
	*Quaternion*		2.15 (0.31)		−0.05 (0.06)	2.28 (0.50)		−0.05 (0.06)	ns
***Uneven Surface***									
*RMS Acceleration*	*Detrending*	*AP*	1.27 (0.22)	1.35 (0.21)	−0.08 (0.09)	1.49 (0.22)	1.65 (0.24)	−0.16 (0.10)	ns
	*Quaternion*		1.39 (0.21)		0.04 (0.08)	1.65 (0.22)		0.00 (0.10)	ns
	*Detrending*	*ML*	1.22 (0.15)	1.25 (0.19)	−0.03 (0.11)	1.15 (0.23)	1.19 (0.22)	−0.04 (0.10)	ns
	*Quaternion*		1.22 (0.16)		−0.03 (0.07)	1.15 (0.24)		−0.04 (0.08)	ns
	*Detrending*	*VT*	2.17 (0.38)	2.29 (0.41)	−0.12 (0.08)	2.38 (0.57)	2.50 (0.54)	−0.12 (0.08)	ns
	*Quaternion*		2.22 (0.38)		−0.07 (0.07)	2.42 (0.55)		−0.07 (0.06)	ns

**NOTE**: ns = difference between the IC3 and Vicon measures (delta) was not significantly different between the older and younger participant groups.

Data are mean (SD) anteroposterior (AP), mediolateral (ML) and vertical (VT) RMS accelerations. Delta represents the difference between the InertiaCube3 and Vicon accelerations.

**Figure 3:**
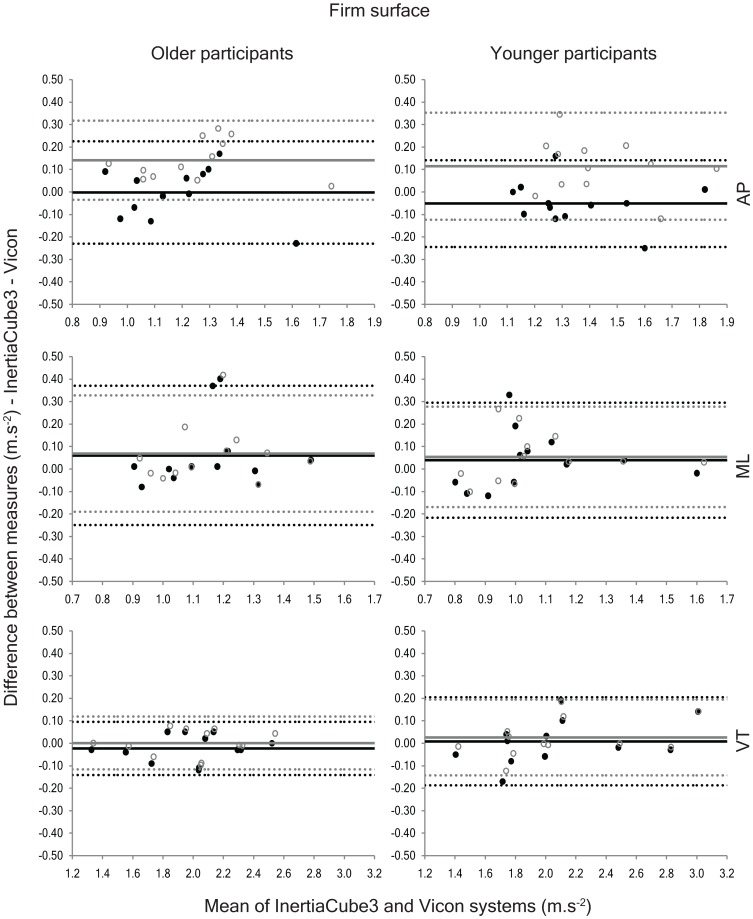
Bland-Altman plots depicting the agreement between the anteroposterior (AP), mediolateral (ML) and vertical (VT) accelerations collected using the InertiaCube3 and Vicon systems while walking on the firm walkway. The solid black circles represent the agreement between the two instruments using the detrending method and the open grey circles show the agreement using the quaternion method. The solid lines on the graphs represent the systematic bias between the two measures and the dashed lines portray the 95% limits of agreement (black = detrending method; grey = quaternion method).

The average difference between the accelerations derived from the IMU and the Vicon system (delta) showed excellent agreement for ML and VT accelerations on the firm walkway using both methods of gravity correction (delta range = −0.02 to 0.06 m.s^−2^ & 0.00 to 0.07 m.s^−2^ for the detrending and quaternion methods, respectively; Figure 3), but the detrending method yielded significantly better results for AP acceleration (delta range = −0.05 to 0.00 m.s^−2^ & 0.11 to 0.14 m.s^−2^ for the detrending and quaternion methods, respectively). These findings were supported by the Bland-Altman analyses (Figure 3), which highlighted a significant positive bias for AP accelerations when the quaternion method was used to compensate for gravitational accelerations on the firm surface. Furthermore, the ICC data (Table 3) indicated that agreement between the Vicon system and the IMU was improved by approximately 13% when AP accelerations were derived using the detrending method on this surface.

**Table 3:** Intraclass correlation coefficients (ICC), standard error of measurement (SEM) and minimal detectable difference (MDD) for the InertiaCube3 and Vicon data.

			Older Participants (n = 12)			Younger Participants (n = 12)		
Surface	Method	Axis	ICC	SEM	MDD	ICC	SEM	MDD
**Firm**	*Detrending*	*AP*	0.84 (0.54–0.95)	0.08 (0.04–0.13)	0.22 (0.12–0.37)	0.88 (0.62–0.96)	0.07 (0.04–0.13)	0.20 (0.11–0.36)
	*Quaternion*		0.76 (−0.06–0.94)	0.11 (0.05–0.23)	0.30 (0.15–0.63)	0.73 (0.10–0.92)	0.11 (0.06–0.20)	0.31 (0.16–0.56)
	*Detrending*	*ML*	0.63 (0.16–0.88)	0.11 (0.07–0.11)	0.31 (0.18–0.47)	0.84 (0.55–0.95)	0.09 (0.05–0.15)	0.25 (0.14–0.42)
	*Quaternion*		0.71 (0.27–0.91)	0.10 (0.06–0.16)	0.27 (0.16–0.43)	0.87 (0.61–0.96)	0.08 (0.05–0.14)	0.23 (0.13–0.40)
	*Detrending*	*VT*	0.98 (0.94–0.99)	0.04 (0.02–0.08)	0.12 (0.07–0.22)	0.98 (0.93–0.99)	0.07 (0.04–0.12)	0.18 (0.10–0.34)
	*Quaternion*		0.99 (0.95–1.00)	0.04 (0.02–0.04)	0.11 (0.06–0.20)	0.98 (0.95–1.00)	0.06 (0.03–0.11)	0.17 (0.09–0.30)
**Compliant**	*Detrending*	*AP*	0.92 (0.50–0.98)	0.07 (0.03–0.17)	0.19 (0.09–0.47)	0.85 (0.14–0.97)	0.10 (0.05–0.24)	0.28 (0.14–0.68)
	*Quaternion*		0.92 (0.40–0.98)	0.07 (0.03–0.18)	0.18 (0.09–0.51)	0.95 (0.84–0.98)	0.06 (0.03–0.10)	0.16 (0.08–0.28)
	*Detrending*	*ML*	0.77 (0.38–0.93)	0.09 (0.05–0.15)	0.25 (0.14–0.41)	0.93 (0.78–0.98)	0.06 (0.03–0.10)	0.16 (0.09–0.28)
	*Quaternion*		0.92 (0.76–0.98)	0.05 (0.09–0.03)	0.14 (0.07–0.24)	0.96 (0.86–0.99)	0.04 (0.02–0.08)	0.12 (0.07–0.22)
	*Detrending*	*VT*	0.96 (0.46–0.99)	0.07 (0.03–0.23)	0.18 (0.08–0.65)	0.98 (0.79–1.00)	0.07 (0.04–0.23)	0.21 (0.10–0.64)
	*Quaternion*		0.98 (0.86–0.99)	0.05 (0.02–0.12)	0.14 (0.07–0.33)	0.99 (0.94–1.00)	0.05 (0.03–0.12)	0.14 (0.07–0.35)
**Uneven**	*Detrending*	*AP*	0.85 (0.32–0.96)	0.08 (0.04–0.18)	0.23 (0.12–0.50)	0.72 (−0.08–0.93)	0.12 (0.06–0.25)	0.34 (0.17–0.68)
	*Quaternion*		0.93 (0.77–0.98)	0.06 (0.03–0.10)	0.15 (0.08–0.28)	0.91 (0.70–0.97)	0.07 (0.04–0.12)	0.19 (0.10–0.34)
	*Detrending*	*ML*	0.79 (0.45–0.93)	0.08 (0.04–0.12)	0.21 (0.12–0.34)	0.90 (0.69–0.97)	0.07 (0.04–0.12)	0.20 (0.11–0.34)
	*Quaternion*		0.90 (0.70–0.97)	0.05 (0.03–0.09)	0.15 (0.08–0.15)	0.93 (0.76–0.98)	0.06 (0.03–0.11)	0.17 (0.09–0.31)
	*Detrending*	*VT*	0.94 (0.16–0.99)	0.10 (0.04–0.36)	0.27 (0.12–1.00)	0.97 (0.41–0.99)	0.10 (0.04–0.42)	0.27 (0.12–1.16)
	*Quaternion*		0.97 (0.69–0.99)	0.07 (0.03–0.22)	0.19 (0.09–0.60)	0.98 (0.78–1.00)	0.07 (0.03–0.25)	0.18 (0.08–0.70)

Data represent the mean (range) ICCs, SEMs and MDDs averaged over three trials and the 95% limits of agreement for the three-dimensional accelerations are presented in brackets. The p-value for all ICCs was p<0.009. AP – anteroposterior; ML – mediolateral; VT – vertical.

**Figure 4:**
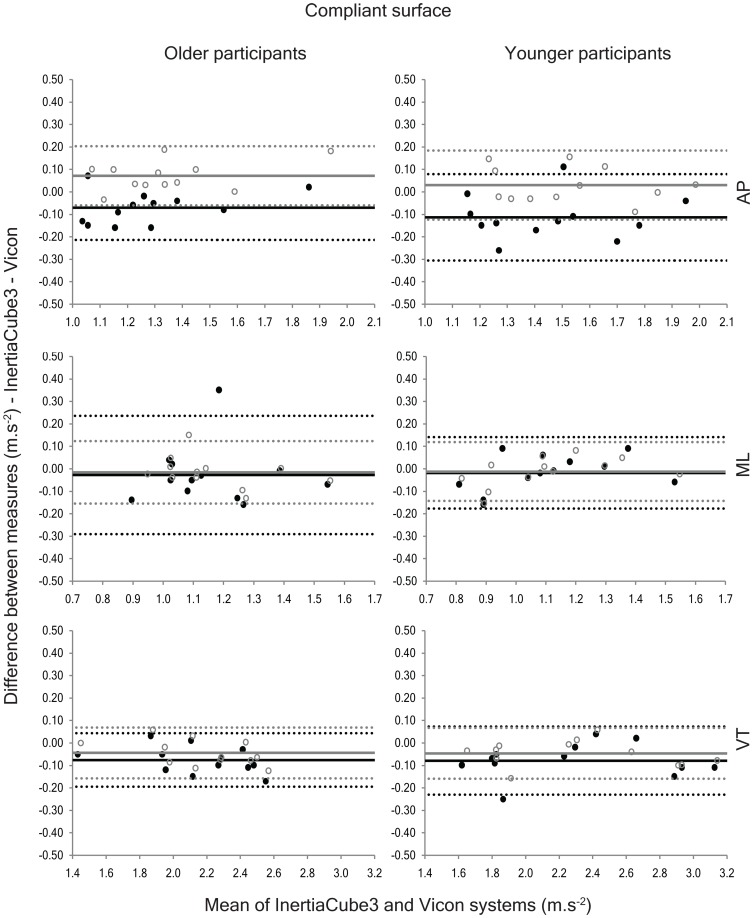
Bland-Altman plots portraying the agreement between the anteroposterior (AP), mediolateral (ML) and vertical (VT) accelerations collected using the InertiaCube3 and Vicon systems while walking on the compliant surface. The solid black circles represent the agreement between the two instruments using the detrending method and the open grey circles show the agreement using the quaternion method. The solid lines depict the systematic bias between the two measures and the dashed lines represent the 95% limits of agreement (black = detrending method; grey = quaternion method).

In contrast, the detrending method produced AP and VT accelerations that were significantly lower than the Vicon system and the quaternion method (Table 2) on the compliant surface and the quaternion method yielded AP accelerations that were significantly greater than the Vicon system and VT accelerations that were significantly lower. Bland-Altman analyses confirmed a significant positive bias for AP accelerations determined using the quaternion method and significant negative biases for VT accelerations calculated via the quaternion method and AP and VT accelerations computed using the detrending method (Figure 4). Although the results showed that the IMU accelerations differed from the Vicon accelerations for both methods of gravity compensation, the quaternion method had consistently better agreement with the Vicon system (ICC range = 0.92 to 0.99) than the detrending method (ICC range = 0.77 to 0.98).

**Figure 5:**
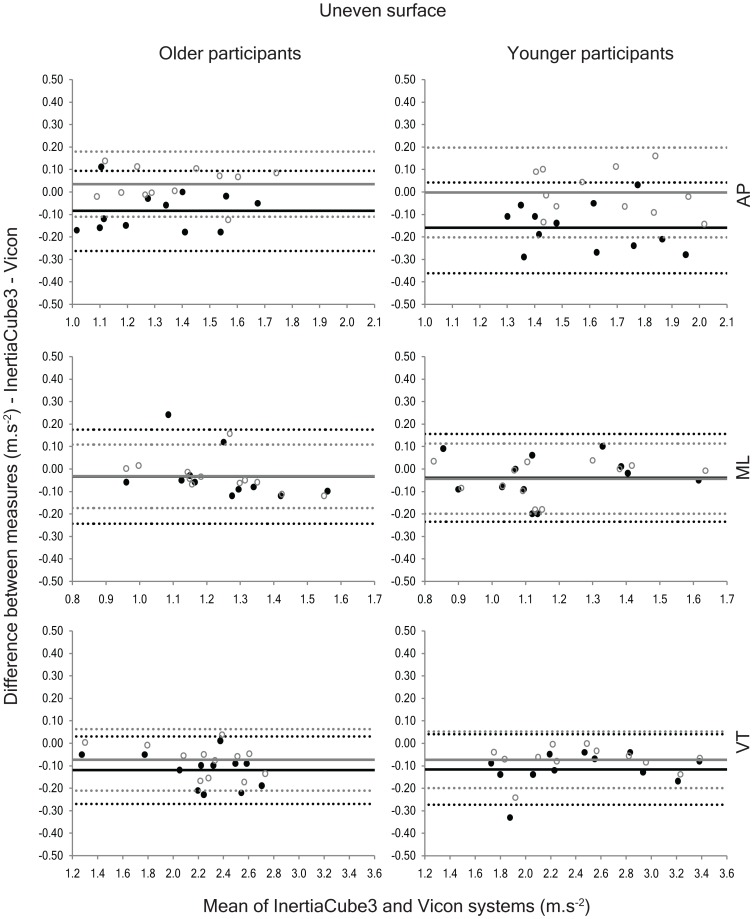
Bland-Altman plots depicting the agreement between the anteroposterior (AP), mediolateral (ML) and vertical (VT) accelerations collected using the InertiaCube3 and Vicon systems while walking on the uneven surface. The solid black circles represent the agreement between the two instruments using the detrending method and the open grey circles show the agreement using the quaternion method. The solid lines represent the systematic bias between the two measures and the dashed lines indicate the 95% limits of agreement (black = detrending method; grey = quaternion method).

On the uneven surface, detrending yielded AP accelerations that were significantly lower than the Vicon system and VT accelerations that were significantly less than both the Vicon and quaternion measures (Table 2). Using the quaternion method, vertical accelerations were also significantly less than the Vicon data, but AP and ML accelerations were not significantly different from the reference system. The assessment of measurement bias supported these findings, highlighting a significant negative bias for AP accelerations when detrending was used, and for VT accelerations when either method of gravity compensation was employed (Figure 5). Nevertheless, the measures derived using the quaternion method more closely approximated the Vicon measures on the uneven surface and this was reflected in the better agreement observed between the quaternion method and the reference system on this surface (ICC range = 0.72 to 0.97 & 0.90 to 0.98 for the detrending and quaternion methods, respectively).

The age of the participants did not influence the agreement between the systems, and the IMU provided accurate acceleration patterns along all three axes while walking on the firm, compliant and uneven surfaces for both groups (Table 3). With the exception of the ICCs reported for ML accelerations for the older group on the firm surface and the AP accelerations presented for the younger participants on the uneven surface, all ICCs were greater than 0.73. These two lower ICC values were both recorded when the detrending method was used to compensate for gravitational acceleration. The SEM and MDD values, were small for both the older (SEM range = 0.04 to 0.11 m.s^−2^ & 0.04 to 0.11 m.s^−2^; MDD range = 0.12 to 0.31 m.s^−2^ & 0.11 to 0.30 m.s^−2^ for the detrending and quaternion methods, respectively) and younger participants (SEM range = 0.06 to 0.12 m.s^−2^ & 0.04 to 0.11 m.s^−2^; MDD range = 0.16 to 0.34 m.s^−2^ & 0.12 to 0.31 m.s-2 for the detrending and quaternion methods, respectively).

## Discussion

This study confirms the concurrent validity of accelerations derived from a light-weight IMU positioned on the trunk (against data collected using a motion analysis system as the gold standard) of younger and older people while walking on firm, compliant and uneven surfaces. A key observation was that trunk acceleration amplitude could be accurately measured using wireless accelerometer technology, even on surfaces that were less stable and predictable and, thus, representative of a real-world setting. Additionally, the results indicated that compensation for gravitational acceleration using the orientation information provided by the IMU was superior to using detrending methods, when the walking surface was compliant or uneven.

Although numerous studies have investigated the accuracy of orientation [22]–[26] and acceleration data [26] derived from three-dimensional IMUs on predictable walking surfaces, this is the first study to explicitly evaluate the concurrent validity of accelerations derived from an IMU while walking on compliant and uneven surfaces. While walking on a treadmill at a preferred speed, excellent agreement has been reported for measures of pitch [23], [25], roll [23], [25] and yaw [23] derived from 3D motion analysis and a trunk-based IMU (all RMS errors≤1.1°). Furthermore, using a series of single-axis accelerometers, gyroscopes and a Vicon motion analysis system, Mayagoitia and colleagues [26] reported excellent agreement for measures of joint orientation, angular velocity and angular acceleration for the lower limb (all RMS error values <7%; all coefficient of multiple correlations >0.98). Similarly high levels of agreement have been reported for gait-related changes in pitch, roll and yaw of the trunk for stroke survivors (all RMS errors ≤1.1°) and people with Parkinson's disease (all RMS errors ≤1.3°) during overground walking [22]. However, slightly poorer agreement was reported for measures of trunk and lower limb orientation during a series of overground walking tasks completed by a single healthy adult (RMS error = 3.6° for both segments) [24]. Collectively, these studies provide support for the use of accelerometers to assess walking under controlled conditions. The current study extends these findings by demonstrating that trunk accelerations can be accurately depicted while walking on more challenging terrains.

The amplitude of trunk accelerations for the older and younger participants were comparable with previous data of younger [11], [15], [17] and older adults [15], [17] on firm and/or irregular walking surfaces. In the present study, when walking on the compliant and uneven surfaces, there was a systematic tendency for the detrended accelerations to be smaller than the accelerations recorded using Vicon (range = −0.02 to −0.16 m.s^−2^), but this was less evident when gravity was subtracted using the quaternion data (range = −0.07 to 0.07 m.s^−2^). The main difference between the two methods was the manner in which gravitational acceleration was accounted for. The detrending method assumed that gravity offsets the data in a constant manner and did not account for gravitational acceleration that potentially violated this assumption. For example, the detrending method may not be appropriate when the orientation of an accelerometer changes in relation to gravity more rapidly than a linear fit of the data can predict. As such, correction for the effects of gravity on a sample-by-sample basis using the orientation information (quaternions) provided by the IMU would be expected to provide a more accurate representation of segmental accelerations and this was confirmed by the present results.

It should be acknowledged that some of the small differences observed between the IMU and Vicon systems in the current study can be explained by the fact that the accelerations were directly measured by the IMU, but were calculated from displacement data for the Vicon system. It is widely recognised that derivation of accelerations from displacement data using the conventional finite difference approach can amplify high-frequency noise present in the displacement data. Although the displacement data collected using the Vicon system were low-pass filtered prior to differentiation, it is important to consider the potential influence on the comparison data. Furthermore, although the findings presented in this study provide support for the validity of gait-related accelerations derived from a trunk-mounted IMU in healthy older and younger adults, additional research is required to determine whether these findings are transferable to other populations.

In summary, this study demonstrates that a light-weight three-dimensional IMU can accurately evaluate trunk accelerations for healthy younger and older adults while walking on firm, compliant and uneven surfaces. Although, the detrending method provides slightly better gravity compensation on the firm surface for AP accelerations derived from this IMU, agreement was improved when gravity was subtracted using the quaternion method on the compliant and uneven surfaces. These findings have important implications for the investigation of postural stability and gait on more challenging surfaces that better approximate real-world environments (e.g. within the home). Future research is needed to examine whether accelerometer technology is suitable to evaluate postural stability and gait during more complex tasks, such as turning and changing direction while walking.
